# Research on the Influence of Informal Employment on Residents’ Happiness in China: Empirical Analysis Based on CLDS Data

**DOI:** 10.3390/ijerph19159085

**Published:** 2022-07-26

**Authors:** Guangyan Chen, Feng Qiu, Xiaowen Dai, Hongxing Lan, Jiahao Song

**Affiliations:** 1 Sichuan Rural Development Research Center, Sichuan Agricultural University, Chengdu 611130, China; 14639@sicau.edu.cn (G.C.); daixiaowen@sicau.edu.cn (X.D.); 13504@sicau.edu.cn (H.L.); songjiahao@sicau.edu.cn (J.S.); 2 Western Rural Revitalization Research Center, Sichuan Agricultural University, Chengdu 611130, China

**Keywords:** informal employment, residents’ happiness, labor market, employment discrimination, impact mechanism

## Abstract

The influence of informal employment on residents’ happiness has gained wide attention around the world. However, few studies focus on this topic in China. Using the 2016 wave of the China Labor Force Dynamics Survey (CLDS) data, we examined the effect of informal employment and its mechanisms on residents’ happiness in China. Our study shows there is a significant negative correlation between informal employment and residents’ happiness in China. Moreover, the correlation between informal employment and residents’ happiness is stronger for residents who are female, migrating, and with a rural household registration. In addition, we investigated possible mechanisms of the effect, including individual income, social respect, unemployment expectations, and social security, and found that informal employment reduces the happiness of residents by widening the gap in unemployment probability and social insurance level among residents.

## 1. Introduction

Aristotle once said: “Happiness is the meaning and the purpose of life, the whole aim, and end of human existence.” Ng [1] also pointed out that happiness is the ultimate goal of people’s rationality and public policy. From the perspective of economic rationality, the pursuit of happiness can at least be regarded as an important goal of life for most residents. However, due to the long-term lack of material resources, we regard the economic growth rate as the ultimate development goal for a long time, ignoring the pursuit of happiness. In terms of function value, happiness as “invisible national wealth” may not only affect individual human capital accumulation [2,3], social relations [4,5], and production efficiency [6,7]. In general, residents’ happiness may also have a profound effect on the economic development, social stability, income distribution, and welfare of a nation. Therefore, it is necessary to conduct in-depth research on the factors affecting residents’ well-being.

Although economic research on happiness started late, the achievements are rich. The topic of the relationship between income and happiness is always one of the most popular research directions in studies. In the article “Does economic growth improve the human lot? Some empirical evidence”, Easterlin found that an increase in a country’s income level did not always improve the national average happiness. In some periods, there was even a reverse relationship between income levels and happiness [8], which is called the “Easterlin Paradox”. Although not all scholars agree with the existence of the “income–happiness paradox” [9,10,11], this “paradox” lays the groundwork for the study of the relationship between income and happiness from the perspective of relative deprivation. For example, a study by Luttmer [12] found that due to the relative increase of surrounding peoples’ income, people who get a controlled or fixed income would have a negative sense of happiness, and the more unequal the personal income received by people, the bigger the gap that exists in the sense of happiness among them [13].

In addition to income, unemployment and inflation are also important factors that affect residents’ happiness. Most scholars believe that unemployment will decrease residents’ happiness. The study by Clark et al. [14] shows that unemployment can significantly reduce the happiness of the unemployed, and the negative impact will increase with the unemployed person’s education level. The research of Alesina et al. [15] further approved that when a country’s overall unemployment rate rises, there will also be a significant reduction in residents’ sense of happiness [15]. Similarly, inflation also has a significant negative impact on residents’ happiness [15,16]. The reason why inflation would reduce residents’ happiness is mainly that people’s current and future income levels may be affected by inflation expectations and the subsequent actual inflation [17,18,19].

Different from the studies we mentioned above, our concern is in China, which has an underdeveloped labor market and long-term large-scale informal employment, and how and to what extent informal employment affects residents’ happiness. As we all know, with the continuous development of various factor markets since the Reform and Opening Up, China’s single employment model has gradually been broken, resulting in the continuous expansion of the scale of informal employment, and has eventually developed into an important form of labor employment. According to an estimate, China’s informal employment rate was only 0.16% in 1978, but in 2013 it rose to 60.2% [20]. In terms of function value, informal employment plays an important role in alleviating employment pressure, reducing poverty, optimizing industrial structure, and establishing and improving the labor market in China [21,22,23,24]. However, informal employment is usually associated with low employment quality characteristics such as lack of social security, no formal employment relationship, low wages, high employment risks, and few development opportunities. Will this form of employment widen the economic and social gap between different groups of people, thereby damaging the happiness of informal workers? This is one of the community-wide concerns.

A growing number of studies have examined the relationship between informal employment and residents’ happiness in the last two decades. In the UK, a seminal study by Taylor [25] showed that temporary employment significantly reduced people’s happiness, and stable employment had a positive effect on improving their happiness in the 1990s. Robone et al. [26] also found that informal employment hurts residents’ happiness, and this impact to a certain extent depends on people’s working time preferences, family conditions, and their employability. Similar evidence of a negative correlation between informal employment and residents’ happiness has been found in several other developed and developing countries, including Japan [27], Canada [28], Sweden [29], Belgium [30], and Bosnia and Herzegovina [31]. However, the informal employment–happiness gradient association is still unclear in the literature, because other studies have found different results. For instance, Bardasi and Francesconi [32] found that typical employment, which includes both temporary and part-time employment schemes, does not cause losses to individual happiness in the UK. Rojas [33] used data from Mexico to examine the relationship and found that there is little difference in happiness between informal workers and formal ones when substantial differences in the socio-demographic and economic characteristics are taken into consideration, as did Schumann et al. [34] in Germany.

In summary, the studies of the relationship between informal employment and residents’ happiness not only have not reached a consensus, but the existing studies are mostly based on the data of developed countries, and the empirical research on a country like China with a large number of informally employed residents is insufficient. In addition, existing studies have seldom investigated the influence of informal employment on the happiness of different characteristics of residents and the channels of informal employment on the happiness of residents.

The purpose of this paper is to estimate the effect of informal employment on residents’ happiness in China and to contribute to the existing literature in several key ways. First, our analyses were based on a large data set from the world’s largest developing country, China. Given rapid economic growth and its accompanying massive labor migration in China, the well-being of tens of millions of special groups, especially the migrant population, rural population, and females are of major importance to public policy in China. If social exclusion and deprivation is a key factor that increases poverty and reduces the happiness of those special groups, informal employment may not only damage the well-being of these special groups but may also reduce the happiness of their family members. Second, we estimated the heterogeneity of associations between informal employment and residents’ happiness. Specifically, we measured differences in the effects by residents’ household registration (rural or urban), residents’ migration situation (immigrant or resident), and resident gender (male and female). Third, we also examined four channels through which informal employment may affect the happiness of residents: (1) annual individual income; (2) social respect; (3) expected unemployment level; and (4) social security level. Finally, we used the strategy of “replacing the explained variable” and “Ordered Probit regressions with Instrumental Variables” (IV-oprobit) to address potential endogeneity in our analyses and checked the robustness of our findings.

This study is structured as follows: Section 2 describes the data and main variables. Next, Section 3 outlines the empirical framework. Section 4 presents regression results. Section 5 discusses the main theoretical and practical contributions of this study to this research field. Finally, Section 6 concludes the paper with policy implications.

## 2. Data and Variables

### 2.1. Data

The data used in this research mainly comes from the “China Labor Force Dynamics Survey (CLDS)”, which has been conducted by the Social Science Survey Center at Sun Yat-sen University in China. The CLDS is a nationwide and authoritative survey of data for labor. The project started in 2012 and has now completed the baseline survey and two rounds of tracking surveys. It adopts multi-stage, multi-level, and probabilistic sampling methods (multistage cluster, stratified, and PPS sampling) in proportion to the size of the labor force, which can meet the need for scientific and reasonable sampling.

We used the latest data of CLDS (2016), since CLDS focuses on the living and working conditions of the Chinese labor force and can truly capture the relationship between informal employment and residents’ happiness. Furthermore, we use the provincial pension insurance coverage rate as an instrumental variable, and the data on the provincial pension insurance coverage rate are mainly from the statistical yearbooks of the provinces, cities, and districts in 2015. We only use the data of laborers aged from 16 to 65. To ensure the balance and comparability of the samples, this study only retains samples whose employment status is “employed”. After excluding samples with missing key data, 4619 valid samples were ultimately retained.

### 2.2. Dependent Variables

The dependent variable in our research is residents’ happiness. According to the content of the CLDS questionnaire, we chose the answers to the question *“In general, do you think your life is happy?”*, which is in the individual questionnaire, to measure the happiness of residents. There are 5 options to this question, where 1 is extremely unhappy, 2 is unhappy, 3 is a bit happy, 4 is happy, and 5 is very happy, which means the levels of residents’ happiness increase as the number increases.

### 2.3. Independent Variables

#### 2.3.1. Informal Employment

The key independent variable in this research is informal employment. In empirical studies in China, whether the employee has official labor contracts or corporate pensions is usually used as the criteria for defining informal employment. Referring to the research of Wang et al. [35], we define residents who have not signed a labor contract and have no pension insurance, including enterprise annuities, provided by enterprises as informally employed, and assign to them a value of 1; otherwise, they are defined as regularly employed and are assigned a value of 0.

#### 2.3.2. Control Variables

Besides informal employment, other factors affect residents’ happiness. Referring to the research of Jackson [36], Mackerron [37], and Sarracino [38], we selected control variables that affect residents’ happiness from the individual level, family level, community level, and province level separately. Control variables of the individual-level include age, the square of age, gender, political status, religious beliefs, household registration type, education level, health condition, and marital status. Family-level control variables include family size (the number of people living in the same family). Community control variables include residential community types. Province-level variables include real GDP growth rate and the minimum wage level.

### 2.4. Descriptive Statistics

Table 1 presents descriptive statistics for the total sample. For the dependent variable, the average resident’s happiness score is 3.878, indicating that there are relatively few residents with severe unhappiness. For the key independent variable, the mean value of informal employment is 0.800, showing that informally employed residents account for the absolute proportion of employed residents.

In terms of control variables, the average values of age and square of age are 38.85 and 1629, respectively. Males account for more than half of the sample (55.4%). Most of the residents have not completed senior middle school, as the mean value of education level is 3.921. More than half of the respondents are married and presently live with their spouses (0.809). The mean value of health condition is 3.904, indicating that residents’ health is relatively good. The number of residents who are members of political groups is very small, and there are very few residents who have religious beliefs. Among the residents, the rural population is slightly higher than the urban population. The average family size is 4.235, indicating that most of the family population in China is already less than 5 people. The mean value of residential community types is 0.613, indicating that the majority of employed residents are employed in urban areas. In addition, Table 1 also separately shows that the average value of the real GDP growth rate and the minimum wage level are 7.910 and 0.255.

## 3. Methodology

Although the maximum likelihood estimation (ML) under the Order Probit (Logit) model is the best way to estimate a dependent variable with a 5-point orderly selection in theory, we still choose to use the OLS method to estimate the impact of informal employment on residents’ well-being. We choose to use the OLS method to estimate the impact of informal employment on residents’ well-being mainly for two reasons. One is that many studies have confirmed that the parameter estimates of OLS regression and Oprobit regression are consistent in direction and significance [39,40,41]; second, the estimated value obtained by OLS has stronger intuitive explanatory power than Oprobit regression. When using the OLS model to estimate the impact of informal employment on residents’ well-being, we also provide Oprobit estimation results to verify the reliability of the OLS estimation results. The basic linear function of residents’ happiness production is specified as follows:

*H_i_* = *α* + *βIE_i_* + *γX_i_*+ *μ_i_* (1)

where *H_i_* is the dependent variable of residents’ happiness. *IE_i_* is the independent variable of informal employment (whether the resident *i* is engaged in informal employment). *X_i_* is a set of control variables, including characteristics of resident, family, and region; and *ε_i_* is an unobserved disturbance term.

## 4. Empirical Results and Analyses

### 4.1. Informal Employment and Residents’ Happiness

Table 1 presents the estimated results of the association between informal employment and residents’ happiness with two specifications, in which the columns from (1) to (4) are the estimated coefficient of OLS regression, and the columns from (5) to (8) are the estimated coefficient of Oprobit regression. Province and community dummies are included in all models, and Huber–White robust standard errors are reported in parentheses.

The estimated results from column (1) to column (4) in Table 2 show a significant negative association between informal employment and residents’ happiness. More specifically, residents participating in informal employment are associated with a 0.085 standard deviation in happiness score growth. This conclusion is consistent with the research conclusion of Wang et al. [27], whose study shows residents engaging in informal employment will significantly reduce their happiness. The Oprobit regression results from column (5) to column (8) are similar to the previous OLS regression results, which also show that informal employment hurts residents’ happiness.

For control variables, male residents’ happiness is relatively lower than female residents, which may be ascribed to those male residents bearing more family financial burdens in China. Resident’s human capital and social capital accumulation are important ways to improve their happiness, as their political status, education level, health condition, and marital status are positively associated with their happiness. Having more family members is positively associated with residents’ happiness. Residents’ age is significantly negatively associated with their happiness, while their age squared is significantly positively associated with their happiness, indicating that the influence of age on residents’ happiness is U-shaped. Religious belief has a significant positive impact on residents’ happiness. This is perhaps because religious beliefs can relieve the residents’ life pressure, thereby enhancing their psychological happiness. The types of household registration have a negative impact on residents’ happiness, but it is not significant. This may be due to the weakening of the linkage between the household registration system and social security in recent years. Meanwhile, our research finds that the type of residential community hurts residents’ happiness. The possible reasons are that residents who live in urban communities have an absolute advantage over those in rural communities, but the former face greater pressures than the latter as the fast pace of city life. In addition, our paper finds that the real GDP growth rate has a significant negative impact on residents’ happiness, possibly because the greater the real GDP growth rate, the greater the social income gap. Finally, our research also finds that the minimum wage level does not promote the happiness of residents, which may be because the minimum wage level in China is still too low to protect the rights and interests of residents.

### 4.2. Heterogeneity of the Relation between Informal Employment and Residents’ Happiness

Investigating the heterogeneous associations between informal employment and residents’ happiness is beneficial to better understanding the effects of the employment policy in China. Therefore, we divided our sample into subgroups by resident’s household registration (rural or urban), resident’s migration status (immigrant or residents), and resident’s gender (male and female). For conciseness, we only used the OLS model to analyze the relationship between informal employment and residents’ happiness in the following subgroup analyses.

The regression estimates of subgroup analyses are presented in Table 3. The results show a larger negative effect of informal employment on the happiness of females than males. One possible reason is the deep roots of patriarchy that lie in Chinese culture, especially in the job market. Compared with males, females engaged in informal employment usually have less salary and a worse employment environment. In terms of residents’ migration, the informal employment marginal utility is substantially larger and more statistically significant for immigrant residents, indicating that immigrant residents suffer more loss of happiness from informal employment than residents. This is possible since these residents who come from other places may be affected by factors such as language, regional culture, social relationships, and regional discrimination, which could increase the negative impact of informal employment on their happiness. In terms of residents’ household registration, the informal employment marginal utility is the largest and statistically significant for rural residents compared to urban residents, showing that informal employment is more harmful to the happiness of residents from rural areas.

### 4.3. Possible Channels of the Relation between Informal Employment and Residents’ Happiness

The previous sections have shown a significant and negative association between informal employment and residents’ happiness. But how does this association arise? In this section, we applied a stepwise approach to identify the mechanisms of the effect, which consisted of two steps: first, we examined the effects of informal employment on possible intermediate factors which may affect residents’ happiness; second, we included intermediate factors that were significant in the first step as independent variables alongside informal employment measure in modeling residents’ happiness outcomes. If intermediate factors in the second step are significantly associated with residents’ happiness outcomes and the negative marginal utility of informal employment is reduced in the estimation, then we can determine the following conclusions that these factors are the mediators between informal employment and residents’ happiness.

Prior studies have demonstrated that informal employment can damage the happiness of residents through the following mechanisms. First, compared with the regularly employed residents, the informally employed residents are relatively deprived of income, which harms the residents’ happiness [42]. The income of informal employees is always significantly lower than that of formal employees [43,44,45]. Second, a lack of respect from the employers may result in a more negative impact on the happiness of the informal employees. Third, job instability and lack of formal and stable labor relations are the basic characteristics of informal employment [46,47], and the increase in informal employment is also an important manifestation of instability in the labor market. Therefore, residents may face a greater risk of unemployment because they are engaged in informal employment, which in turn reduces their happiness. Fourth, a high level of social security can significantly improve people’s happiness [48]. However, the existence of “difficulties to adapt to a unified social security system” and “employers not fulfilling the obligation to purchase informal workers’ insurance” makes it difficult for informal workers to enjoy adequate insurance [49,50]. Low levels of social insurance or no social insurance may jeopardize the happiness of informal workers.

We examined four possible channels through which informal employment affects residents’ happiness in China: individual income, social respect, unemployment expectations, and social security. First, the resident’s after-tax salary income (unit: yuan) was used to measure the individual income. Second, we generated one categorical variable regarding social respect from CLDS questionnaires: “is the meaning or value of your current job to be respected?” There are 5 options to this question, where 1 means extremely agree with this view, 2 means comparatively agree with this view, 3 means agree with this view, 4 means comparatively disagree with this view, and 5 means extremely disagree with this view. Third, how likely is it that residents will encounter unemployment in the next five years (1 = Very likely, 2 = More likely, 3 = Less likely, 4 = Very unlikely, 5 = Do not know) was as the substitute variable for unemployment expectations. Fourthly, we calculated the number of residents’ insurance (including pension, medical insurance, unemployment insurance, work injury insurance, maternity insurance, and housing fund) separately and used it as a substitute variable for social security. The OLS regression models were used to estimate these relationships, and all estimated results were reported in Table 4 and Table 5.

Table 4 presents the results of the relationships between informal employment and individual income, social respect, unemployment expectations, and social security. The results showed that informal employment is significantly and negatively associated with individual income. Compared with the formally employed, the informally employed suffer from income deprivation. On average, the individual income of residents participating in informal employment is 40.1 percent lower than that of residents in formal employment. In terms of social respect, an insignificant and positive association between informal employment and social respect was reported. In terms of unemployment expectations, the results showed that informal employment is significantly and positively associated with unemployment expectations, indicating that the unemployment rate of informal employment residents is expected to be higher than that of formal employment residents. Specifically, informal employment residents are 17.6% more likely to expect to be unemployed than formal employment residents. In addition, our findings also reported that informal employment hurts social security. On average, the amount of social insurance purchased for informally employed residents would be 119.8% lower than for formally employed residents.

After establishing significant associations between informal employment and individual income, unemployment expectations, and social security, we extended the model of informal employment and residents’ happiness with the inclusion of significant intermediate factors in step one (Table 5). The estimates showed that the coefficient of informal employment decreases with the inclusion of unemployment expectations, which is significantly and negatively associated with residents’ happiness, indicating that unemployment expectations are one significant factor that affects residents’ happiness. Furthermore, social security is significantly correlated with residents’ happiness outcomes, displaying that social security is another important factor affecting residents’ happiness. Meanwhile, the estimates showed that the coefficient of informal employment increases with the inclusion of individual income, which is insignificantly but negatively associated with residents’ happiness, indicating that individual income is not a channel through which informal employment affects residents’ happiness, possibly because the income of some informal employment groups in China today is higher than that of formal employment groups. To sum up, our analyses indicated that unemployment expectations and social security are the main linkage factors between informal employment and residents’ happiness in China. In addition to this, informal employment mainly reduces the happiness of residents by widening the gap in unemployment probability and social insurance level among residents.

### 4.4. Robustness Test

Although the estimated result in the previous sections shows that informal employment has a significant negative impact on residents’ happiness, the robustness of this research conclusion has not been verified. In this section, we applied two approaches to check the robustness of our findings. First, we replaced the dependent variable “residents’ happiness” with the “residents’ life satisfaction”, and then we estimated the relationship between informal employment and residents’ life satisfaction. Second, we further employed OLS regression with an instrumental variable to address the potential endogeneity in our analysis.

As we all know, except happiness from subjective feelings, life satisfaction and ladder-of-life are commonly used to measure residents’ happiness, and these measuring variables have a high degree of substitution. Therefore, we replaced the dependent variable “residents’ happiness” with the “residents’ life satisfaction”, and then estimated the relationship between informal employment and residents’ life satisfaction to verify the robustness of the previous estimation results. Combining the research needs and the characteristics of the CLDS questionnaire, we selected question “Generally speaking, are you satisfied with your living conditions?” from the CLDS 2016 individual questionnaire in the robustness analysis as a proxy variable for residents’ happiness. The question options of residents’ life satisfaction are 1–5, where 1 is extremely dissatisfied, and 5 is very satisfied. That is, life satisfaction increases as the value of the question option increases.

Column (1) in Table 6 present marginal utility estimates of informal employment on residents’ life satisfaction. From column (1) in Table 6, we can see that informal employment has a significant negative impact on residents’ life satisfaction, indicating that the impact of informal employment on residents’ happiness is robust.

Although a set of variables that may correlate with residents’ happiness were controlled for in the previous models, potential endogeneity needs to be considered. On the one hand, the dependent variables and independent variables are, very likely, influenced by multiple variables at the same time. For example, family structure and resources may simultaneously affect informal employment and residents’ happiness. Residents with elderly parents and young children who need support may be more likely to participate in informal employment and have a bad sense of happiness. It is also possible that more resourceful families have better capabilities and a higher tendency to participate in formal employment and to have a better sense of happiness. On the other hand, when estimating the relationship between informal employment and residents’ happiness, it is likely to omit important variables, which may also lead to deviations in the estimation results of our paper.

To address these problems, we employed OLS regression with an instrumental variable to address the potential endogeneity in our analysis (the average level of informal employment at the county level as an instrumental variable). Table 6 reports the estimated results of IV-2SLS. In column (2) of Table 6, the *p* value of the average level of informal employment at the county level is highly significant. That is, there is sufficient correlation between the instrumental variables and the endogenous explanatory variables. In addition, the Corr *p*-Value is highly significant, indicating that the instrumental variables meet the exogenous requirements. From column (3) in Table 6, we can see that informal employment has a significant negative impact on the residents’ happiness after alleviating the endogenous problem of the model, which confirms that the regression results in the previous sections are robust. In a word, informal employment does significantly damage residents’ happiness.

## 5. Discussion

This paper contributes to research on the effect of informal employment on residents’ happiness in China by using data from the China Labor Force Dynamics Survey. We not only directly investigated the association between informal employment and residents’ health, but also analyzed the heterogeneity and possible channels of the effect and discussed and tested the possible endogeneity problem.

The results of our study show that residents participating in informal employment are significantly associated with worse happiness status in China. This is consistent with the conclusion of Wang et al. (2020), Taylor (2002), and Robone et al. (2011), whose studies directly or indirectly showed the negative causation between informal employment and residents’ happiness. Our research provides a piece of new evidence for the above negative causation and is of benefit to advancing the current understanding of the relationship between employment and residents’ welfare in developing countries. Therefore, in developing countries with an undeveloped social security system employment market, it is necessary to take measures to guide the development of informal employment, thus ensuring the welfare of residents. In addition, the present study encourages future studies to give adequate attention to the heterogeneity of informal and formal employment in developing countries in order to draw more scientific and realistic conclusions.

We also find that the above negative effect is more obvious when the case is related to females, immigrants, and people with rural household registration. The possible reasons for this are as follows: (1) due to the deep roots of patriarchy which lie in Chinese culture, especially in the job market, compared with males, females engaged in informal employment usually have less salary and a worse employment environment. (2) Residents who come from other places may be affected by factors such as language, regional culture, social relationships, and regional discrimination, which could increase the negative impact of informal employment on their happiness. (3) The dual household registration system in China directly affects the living welfare of residents with different household registrations. Residents with rural household registration cannot enjoy the same social welfare even if they are engaged in the same work as residents with urban household registration [51]. Therefore, government departments must take corresponding measures to protect the interests of women, immigrants, and residents with rural household registration.

For the channels through which informal employment affects residents’ happiness, we tested mechanisms, including individual income, social respect, unemployment expectations, and social security. We found that informal employment is significantly and negatively associated with individual income and social security, and, in contrast, informal employment is significantly and positively associated with residents’ unemployment expectations. Additionally, there is no significant causation between informal employment and social respect. Furthermore, the results of extended models show that only the unemployment expectations and social security of residents are the main linkage factors between informal employment and residents’ happiness. Therefore, according to our research, informal employment mainly reduces the happiness of residents by widening the gap in unemployment probability and social insurance level among residents.

This research has a certain contribution and policy influence on happiness research, but there is still room for improvement. First, we only use the sense of happiness and life satisfaction to measure residents’ happiness, which may not be comprehensive enough, because residents’ happiness includes many aspects. Second, informal employment included many aspects, and we only define informal employment from the labor aspect of pension provided by enterprises, which may not fully represent informal employment. Meanwhile, due to data limitations, we cannot rule out the existence of voluntary informal employment and forced informal employment when taking the labor contract or pension insurance provided by enterprises to define informal employment. Third, the influence of informal employment on residents’ happiness may not be limited to individual income, social respect, unemployment expectations, and social security, so there may be still many mechanisms that we have not tested.

## 6. Conclusions

In conclusion, residents participating in informal employment are significantly associated with worse happiness status in China, because informal employment widens the gap in unemployment probability and social insurance level among residents, and the negative effect is more obvious when the case is related to females, immigrants, and people with a rural household registration.

Our findings have several implications for the improvement of employment policy and residents’ happiness protection. First, the government may consider urging employers to sign legal labor contracts with employees to regulate the employer’s behavior in recruiting and employing people, thereby protecting employees’ legitimate rights and interests, such as salary level, leisure time, and job stability. A large number of studies have shown that the inability of workers to sign labor contracts with employers is an important reason for the unstable work of informal employees. Without the protection of labor contracts, informal workers may be dismissed at will and face the risk of unemployment at any time. Not only that, without the protection of labor contracts, informal employees are more likely to face wage arrears, excessive working hours, poor working environment, and difficulty maintaining personal dignity, which seriously damages the happiness of the workers. Therefore, the government may consider adopting coercive measures to increase punishments for employers who violate the regulations. Only when a standardized labor contract is signed under the current legal framework can the rights and interests of the employees be protected, and their happiness can be improved as well.

Second, the government may consider issuing policies to encourage enterprises to increase the level of social security for informal workers. Our research shows that many employers fail to pay social insurance for their employees, leaving informal workers out of the social security system, which in turn reduces the happiness of informal workers. Therefore, while establishing a sound social security system and advocating universal participation, the government should also strengthen the supervision of residents’ participation in social security and ensure that enterprises pay social insurance for employees. In addition, the government also should encourage enterprises to establish supplementary insurance for employees based on the actual situation.

Third, the government could formulate special laws and regulations against discrimination in the labor market. As we all know, females, non-locals, and agricultural residents engaging in informal employment have a greater loss of happiness, as they suffer more serious employment discrimination in the informal job market. To reduce group discrimination in the labor market, the government should improve laws and regulations and formulate special laws against employment discrimination to ensure equal employment for different workers. For violations of employment discrimination laws, penalties should be increased, and legal responsibilities should be investigated by the law.

## Figures and Tables

**Table 1:** Variable definitions and descriptive statistical results.

Variable	Description or Definition	Mean	S.D.
Residents’ happiness	1: Extremely unhappy; 2: Unhappy; 3: A bit happy; 4: happy; 5: Very happy	3.878	0.875
Informal employment	1: Yes; 0: No	0.800	0.400
Age	Continuous variable	38.85	10.94
Square of age	Continuous variable	1629	868.7
Gender	1: Male; 0: Female	0.554	0.497
Political status	1: Chinese Communists; 0: Others	0.163	0.369
Religious belief	1: Have religious beliefs; 0: No religious belief	0.107	0.309
Household registration type	1: Rural household registration; 0: Others	0.517	0.500
Education level	1: Never went to school; 2: Primary school; 3: Junior high school; 4: Senior middle school; 5: Junior college; 6: Undergraduate; 7: Master; 8: Doctor	3.921	1.355
Health condition	1: Extremely unhealthy; 2: Unhealthy; 3: A bit healthy; 4: Healthy; 5: Very healthy	3.904	0.818
Marital status	1: Married; 0: Others	0.809	0.393
Family size	Continuous variable	4.235	1.900
Residential community types	1: Urban community; 0: Rural community	0.613	0.487
Real GDP growth rate	Continuous variable	7.910	1.262
The minimum wage level	The ratio of the actual minimum wage to the average wage of urban employees	0.255	0.0285

**Table 2:** Informal employment and residents’ happiness.

Variable	OLS				Oprobit			
	(1)	(2)	(3)	(4)	(5)	(6)	(7)	(8)
Informal employment	−0.192 ***	−0.081 **	−0.083 ***	−0.085 ***	−0.235 ***	−0.108 **	−0.110 **	−0.114 ***
	(0.031)	(0.032)	(0.032)	(0.032)	(0.040)	(0.043)	(0.043)	(0.043)
Age		−0.032 ***	−0.031 ***	−0.030 ***		−0.040 ***	−0.038 ***	−0.038 ***
		(0.010)	(0.010)	(0.010)		(0.012)	(0.012)	(0.012)
Square of age		0.000 ***	0.000 ***	0.000 ***		0.001 ***	0.000 ***	0.000 ***
		(0.000)	(0.000)	(0.000)		(0.000)	(0.000)	(0.000)
Gender		−0.069 ***	−0.070 ***	−0.071 ***		−0.095 ***	−0.097 ***	−0.098 ***
		(0.026)	(0.026)	(0.026)		(0.034)	(0.034)	(0.034)
Political Status		0.122 ***	0.121 ***	0.123 ***		0.163 ***	0.163 ***	0.166 ***
		(0.035)	(0.035)	(0.035)		(0.048)	(0.048)	(0.048)
Religious Belief		0.10 1 **	0.100 **	0.105 **		0.131 **	0.131 **	0.137 **
		(0.041)	(0.041)	(0.041)		(0.054)	(0.054)	(0.055)
Household registration type		−0.030	−0.041	−0.057		−0.036	−0.050	−0.073
		(0.031)	(0.031)	(0.035)		(0.040)	(0.041)	(0.046)
Education Level		0.070 ***	0.071 ***	0.072 ***		0.089 ***	0.091 ***	0.092 ***
		(0.012)	(0.012)	(0.012)		(0.016)	(0.016)	(0.016)
Health Condition		0.257 ***	0.259 ***	0.259 ***		0.334 ***	0.336 ***	0.337 ***
		(0.017)	(0.017)	(0.017)		(0.023)	(0.023)	(0.023)
Marital Status		0.298 ***	0.284 ***	0.285 ***		0.378 ***	0.359 ***	0.362 ***
		(0.041)	(0.042)	(0.042)		(0.053)	(0.054)	(0.054)
Family size			0.014 *	0.013 *			0.019 **	0.017 *
			(0.007)	(0.007)			(0.010)	(0.010)
Residential community types				−0.053				−0.073 *
				(0.034)				(0.044)
Real GDP growth rate				−0.038 ***				−0.056 ***
				(0.011)				(0.015)
The minimum wage level				0.115				0.164
				(0.448)				(0.596)
Constant	4.032 ***	3.029 ***	2.950 ***	3.261 ***				
	(0.027)	(0.207)	(0.212)	(0.253)				
*p* Value	0.000	0.000	0.000	0.000	0.000	0.000	0.000	0.000
Observations	4335	4335	4335	4335	4335	4335	4335	4335
R-squared	0.008	0.097	0.098	0.102				
Pseudo R^2^					0.003	0.041	0.041	0.043

Note: *** *p* < 0.01, ** *p* < 0.05, * *p* < 0.1, Robust standard errors in parentheses.

**Table 3:** Regression results of sub-sample.

Variable	(1)	(2)	(3)	(4)	(5)	(6)
	Rural-Household-Registration Residents	Urban-Household-Registration Residents	Floating Population	Local Resident	Male Residents	Female Residents
Informal employment	−0.171 ***	−0.060	−0.222 ***	−0.062 *	−0.064	−0.107 **
	(0.064)	(0.037)	(0.086)	(0.034)	(0.042)	(0.049)
Control variable	YES	YES	YES	YES	YES	YES
Constant	YES	YES	YES	YES	YES	YES
*p* Value	0.000	0.000	0.000	0.000	0.000	0.000
R-squared	0.091	0.106	0.101	0.101	0.110	0.097
N	2242	2093	745	3590	2400	1935

Note: *** *p* < 0.01, ** *p* < 0.05, * *p* < 0.1, Robust standard errors in parentheses.

**Table 4:** Channels of informal employment effect on residents’ happiness.

Variables	Individual Income	Social Respect	Unemployment Expectations	Social Security
Informal employment	−0.401 ***	0.018	0.176 ***	−1.198 ***
	(0.075)	(0.030)	(0.046)	(0.063)
Control variables	YES	YES	YES	YES
*p*-value	0.000	0.000	0.000	0.000
R-squared	0.079	0.040	0.082	0.432
Observations	4335	4335	4335	4335

Note: *** *p* < 0.01, Robust standard errors in parentheses.

**Table 5:** Expanded model of informal employment and residents’ happiness.

Variables	(1)	(2)	(4)	(5)	(6)
Informal employment	−0.085 ***	−0.088 ***	−0.073 **	−0.059 *	−0.051
	(0.032)	(0.032)	(0.032)	(0.033)	(0.033)
Individual income		−0.007			−0.009 *
		(0.005)			(0.005)
Unemployment expectations			−0.072 ***		−0.071 ***
			(0.011)		(0.011)
Social security				0.022 ***	0.022***
				(0.008)	(0.008)
Control variables	YES	YES	YES	YES	YES
*p*-value	0.000	0.000	0.000	0.000	0.000
R-squared	0.102	0.102	0.111	0.103	0.113
Observations	4335	4335	4335	4335	4335

Note: *** *p* < 0.01, ** *p* < 0.05, * *p* < 0.1, Robust standard errors in parentheses.

**Table 6:** Results of the robustness test.

Variable	(1)	(2)	(3)
	Life Satisfaction	Informal Employment	Residents’ Happiness
Informal employment	−0.126 ***		−0.342 ***
	(0.034)		(0.126)
Average level of informal employment at county level		0.760 ***	
		(0.045)	
Control variable	YES	YES	YES
IV T Value		17.050	
IV *p* Value		0.000	
R-squared	0.100	0.215	0.090
*p* Value	0.000	0.000	0.000
N	4335	4335	4335

Note: *** *p* < 0.01, Robust standard errors in parentheses.

## Data Availability

Data supporting the conclusions of this article are included within the article. The dataset presented in this study are available on request from the corresponding author (2020208003@stu.sicau.edu.cn).
